# A systematic review of the application of Wilson and Cleary health-related quality of life model in chronic diseases

**DOI:** 10.1186/s12955-017-0818-2

**Published:** 2017-12-11

**Authors:** Adedokun Oluwafemi Ojelabi, Yitka Graham, Catherine Haighton, Jonathan Ling

**Affiliations:** 1 Department of Pharmacy, Health and Wellbeing, University of Sunderland, Sunderland, UK; 2 University of Ibadan, Ibadan, Nigeria; 3 Department of Social Work, Education and Community Wellbeing, Northumbria University, Northumbria, UK; 4 Institute of Health and Society, Newcastle University, Newcastle, UK

**Keywords:** Health-related quality of life, Conceptual model, Causal relationships, Chronic diseases

## Abstract

**Background:**

A conceptual model approach to clarify the elements of health-related quality of life (HRQL), their determinants and causal pathways is needed to aid researchers, health practitioners and policy makers in their bid to improve HRQL outcomes in patients. The aim of this systematic review was to appraise empirical evidence on the performance of the Wilson and Cleary Model of HRQL.

**Methods:**

We conducted a search of MEDLINE, Science Direct, PsyARTICLES and CINAHL databases to identify articles that used Wilson and Cleary model to examine HRQL in chronic diseases. A narrative synthesis was employed in the review of the articles.

**Results:**

Evidence supports linkages between adjacent concepts and between non-adjacent concepts of the Wilson and Cleary model indicating that in practice there is a need to examine relationships among constructs - or to consider interventions in terms of - both with and without mediators. Symptoms status has the highest magnitude of relative impact on health-related quality of life.

**Conclusion:**

The Wilson and Cleary model demonstrated good features suitable for evaluating health-related quality of life in chronic diseases.

## Background

Health-related quality of life (HRQL) is an important outcome measure in clinical trials and health research. The concept includes measures of patient and social perspectives regarding the impact of illness in order to improve treatment efficacy, safety and shared decision-making [1–3]. Examining the construct of HRQL has become important because it focuses on components of well-being, which are affected by progressive changes in health status, health care and social support [4].

Patients living with chronic illnesses such as sickle cell disease, HIV/AIDs, chronic obstructive pulmonary disease, arthritis and obesity have been found to have impaired HRQL [5]. HRQL has thus become an important construct in the evaluation of the effect of a disease and its management. However, there has been lack of agreement on the definitions and dimensions of HRQL which could guide standard measurements and research that could lead to a more detailed understanding of the concept. Nonetheless, there are three identified areas of consensus, that first, HRQL is a multidimensional construct encompassing symptoms of diseases, treatment side effects, general perception of health status and life satisfaction [6]; second, the assessment of HRQL is subjective based on self-report termed patient-reported outcomes (PROs) [3, 7] and third, research on HRQL should be based on conceptual models [8, 9]. Such models would enhance the understanding of the relationships and linkages among dimensions of HRQL which in turn could facilitate the design of protocols for optimal care.

Wilson and Cleary [10] have proposed a conceptual model to integrate clinical and psychosocial approaches to health care. Their model links the biological and physiological (objective health) variables to the measure of HRQL or subjective health constructs. This link was made to move research on HRQL from the traditional descriptive methods to models, so that causal relationships among the components could be investigated and clarified. Knowing the proximate causes of HRQL in a disease population would help target rather than just monitor the improvement of HRQL in clinical trials.

The Wilson and Cleary model is the most widely cited conceptual framework of HRQL [8, 9, 11]. In a systematic review of health-related quality of life models, Bakas and colleagues [8] showed that the Wilson and Cleary model was unique to HRQL, adequate, clear and consistent and could be applied to all individuals irrespective of age, health and disease conditions as well as culture. They further showed that the Wilson and Cleary model could generate hypotheses to provide clinicians with a broader view of HRQL beyond just biological factors and symptoms, and that the model ‘makes sense’ for real world application. The model focusses on relationships among different domains of health by proposing a linear sequence of causal links along a causal pathway which begins with the bio-physiological level moving along the causal pathway outward to the subjective level and the interaction of the individual as a social being.

The Wilson and Cleary model presents a taxonomy of patient outcomes categorised into five underlying health concepts and proposes specific causal links between these health concepts. Their underlying assumptions are that understanding relationships among these concepts will inform the design of optimally effective clinical interventions ([9] The five health concepts described in the model are biological and physiological factors, symptoms status, functioning, general health perceptions and overall quality of life.

The biological and physiological factors focus on the functioning of cells, organs and organ systems. The clinical factors include factors that generally affected health but are mediated by changes in cells, organs or organ systems functions. The next point on the continuum is symptoms status which has been described as a patient’s perception of an abnormal physical, emotional or cognitive state [10]. The complexity of relationship between biological and physiological factors and symptoms is underlined by the fact that some physiological abnormality may not immediately produce symptoms while some symptoms such as depression may not be clinically traceable to physiologic abnormality [10]. Following symptoms is functional status which is reflected in the ability of the individual to perform specific tasks such as climbing the stairs. The next link is the general health perceptions, a subjective rating that integrates all the previously mentioned health concepts and others such as mental health and is followed by the overall or global health-related quality of life at the end of the continuum [10]. Arrows in the model depict dominant causal associations. Reciprocal relationships are implied but not shown. The possibility of bidirectional relationship has also been suggested [10] but not indicated.

Empirical evidence from studies that have used the Wilson and Cleary model is needed to establish patterns of relationship and their consistency. Integrating results of empirical studies onto the model will reveal the features and performance of the model and enhance our understanding of patterns of relationships and effects of mediators thereby increasing the information available to health researchers and practitioners. Furthermore, understanding the relative importance of each of the concepts with respect to their effects on the overall quality of life may be useful in future research. This paper reports a systematic review of literature on the application of Wilson and Cleary’s model in chronic diseases to examine the paths and pattern of relationships of the concepts as well as determine their relative importance. We aimed to answer three important research questions:

1. Does empirical evidence show the causal relationship of the dominant concepts as proposed in the Wilson and Cleary model?
2. Does the Wilson and Cleary model follow a strictly linear unidirectional path?
3. What is the relative effect of each latent factor?

## Methods

This study followed the format of the Preferred Reporting Items for Systematic Reviews and Meta-Analysis (PRISMA) statement [12]. The electronic databases searched consisted of Science Direct, MEDLINE, CINAHL and PsyARTICLES. The search term used was “Wilson and Cleary” (free text). Further related search terms such as, “Wilson and Cleary model”, “Wilson and Cleary conceptual model”, “(Health-related quality of life OR HRQL OR HRQOL) AND (Wilson and Cleary OR Wilson and Cleary model)”, were also used, but did not yield any additional studies. The search covered a period from 1995 (when the model was published) to December 2016.

### Inclusion criteria:

- Chronic disease
- Articles published in English language
- HRQL measured with validated instruments
- Empirical study
- Wilson and Cleary model was used or tested
- Peer-reviewed articles with full-text accessible.

### Exclusion criteria:

- Articles based on instrument development
- Articles that did not apply the model

The titles and abstracts of retrieved articles were reviewed by AO for eligibility and selected based on the inclusion criteria. The selection was validated by JL. Full-texts of articles were reviewed for inclusion by AO, JL and YG validated these and also agreed on the five articles that were hand-searched for inclusion in the study.

### Quality assessment of selected articles

The Quality Assessment Tool for Quantitative Studies designed by the Effective Public Health Practice Project (EPHPP) was used to evaluate the quality of included articles [13]. The EPHPP tool was designed to assess quality of observational and clinical studies. The tool was used to rate each article on a three-point scale (strong, moderate and weak) in six components: selection bias, study design, confounders, blinding, data collection methods, and withdrawal and drop-outs. A global rating was allocated to each study.

### Data extraction, synthesis and analysis

Standardised data extraction form was used to extract data from the included studies by AOO, these were reviewed independently by YG and JL. Information extracted from each article included author, year of publication disease, study design, measures of latent factors, study aim, outcome of study and percentage of variance explained by the model. Articles selected had the primary objective of testing or applying the Wilson and Cleary model in the disease population. Most of the study designs were cross sectional (77%), heterogeneity was not formally calculated as meta-analysis was not performed.

Three research questions proposed for this study were to evaluate linearity and non-linearity of relationships and the effects of predictor variables on HRQL. Linearity was evaluated based on direct causal links between the concepts along the path of continuum proposed by Wilson and Cleary [10]. Non-linearity was evaluated based on significance of paths between non-adjacent variables - that is we establish that there is non-linearity if the effects between non-adjacent variables were significant so that paths between adjacent and non-adjacent variables were allowed [14]. The magnitude of the influence of each of the variables was also examined to evaluate their relative effects on HRQL.

## Results

**Fig. 1:**
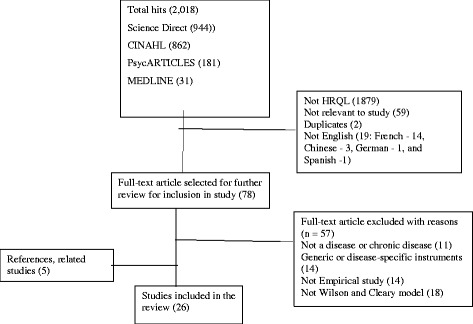
Flow chart of study selection procedure

The initial search yielded a total of 2018 full text peer reviewed articles (Fig. 1). Duplicates were removed and articles were screened on titles and abstracts. The full-texts of the selected 78 articles were screened. Of these, 59 articles were excluded: 14 because they were based on instrument development, 18 did not apply the model, 14 were not empirical studies and 11 could not be categorised as focusing on chronic disease. Five additional articles were added through searching of reference lists of the selected studies. The total number of articles reviewed was 26 [15–40]. The flow chart of the included studies is displayed in Fig. 1.

### Characteristics of studies reviewed

The 26 studies took place in 15 countries with one study based across four countries in Sub-Saharan Africa (Botswana, Lesotho, South Africa and Swaziland) (Table 1). The other countries were: USA (*n* = 9), Norway (*n* = 3), Canada (*n* = 2), Netherlands (*n* = 3), Thailand (*n* = 2), UK (*n* = 1), France (n = 1), Austria (n = 1), Sweden (n = 1), Brazil (n = 1) and Hong Kong (n = 1).

**Table 1:** Quality assessment of included studies

Author	Selection bias	Study design	Confounding	Blinding	Data collection	Withdrawal and drop-out	Overall quality
Ade-Oshifogun	1	3	1	2	1	2	**2**
Arnold	1	2	1	1	1	2	**2**
Baker	1	1	1	1	1	2	**1**
Brunault	1	2	2	1	1	1	**1**
Carlson	1	2	3	2	1	2	**2**
Cosby	1	3	3	2	1	1	**3**
Eilayyan	1	2	2	2	1	2	**2**
Halvorsrud	1	1	2	2	1	1	**2**
Heo	1	1	1	1	1	1	**1**
Hofer	1	2	1	1	1	1	**1**
Kanters	1	2	1	1	1	1	**2**
Krethong	1	3	1	2	1	2	**2**
Mathisen	1	1	2	1	1	1	**1**
Mayo	1	2	3	2	1	1	**2**
Nokes	1	3	2	1	1	1	**2**
Phaladze	1	3	2	2	1	2	**2**
Portillo	1	3	1	2	1	2	**2**
Saengsiri	1	1	2	3	1	1	**2**
Santos	1	1	1	1	1	2	**2**
Schulz	2	2	1	1	1	2	**2**
Shiu	1	3	1	2	1	1	**2**
Sousa (1999)	1	3	1	2	1	2	**2**
Sousa (2006)	1	2	2	2	1	2	**2**
Ulvik	1	3	2	2	1	2	**2**
Wettergren	2	2	1	1	1	2	**2**
Wyrwich	1	2	1	1	1	2	**1**

Note: 1 = low risk of bias, 2 = moderate risk of bias and, 3 = high risk of bias

Thirteen different types of disease were studied: heart failure/surgery (*n* = 5), HIV/AIDS (*n* = 6), coronary artery disease (n = 3), oral health disease (*n* = 2), obesity (n = 2), chronic obstructive pulmonary disease (n = 2) and asthma, diabetes, Hodgkin’s lymphoma, kidney, Pompe disease, generalised anxiety disorder and stroke (all *n* = 1). Study designs were either cross sectional (*n* = 20) or longitudinal (*n* = 6). The total number of participants was 11,849, with a mean age of 50.5 years; 43.7% were female.

### Quality assessment

Six studies were rated as strong (23%), 19 studies had moderate rating (73%) and one study had a weak rating (Table 1).

### Measures

#### Generic instruments

The most widely used instrument was the Medical Outcome Survey (MOS) Short Form 36 (SF-36) [41], in both the full form SF-36 (*n* = 10) and the shorter form 12 [42], SF-12 (*n* = 2). Components of the instrument were used to measure symptoms status (*n* = 3), functional status (*n* = 7), general health perceptions (*n* = 9) and global HRQL (n = 2). Other generic instruments used were the Sickness Impact Scale (SIS), Patient Health Questionnaire (PHQ-9) [43] to measure depression, Beck’s Depression Index (BDI) [44] and the Hospital Anxiety and Depression Scale (HADS) [45].

#### Disease-specific instruments for HRQL

Disease-specific HRQL instruments used in the studies included the MacNew Heart Disease Quality of Life [24], Minnesota Living with Heart Failure Questionnaire (MLFHQ) and the New York Heart Association (NYHA) classification to measure the global HRQL in heart failure [23, 26, 38]. The HIV/AIDS-Targeted Quality of Life (HAT-QoL) instrument [29–31] for HIV/AIDs populations while the Quality of Life, Obesity and Dietetics (QOLOD) [18], Oral Health Impact Profile (OHIP-14) [17, 33] were used in diabetes and oral health populations respectively.

### Analytical tools

In modelling the data (Table 2), different analytical tools were used: stepwise/hierarchical regression (*n* = 3), linear mixed model/multiple regression (*n* = 4), logistic regression and/or partial correlation (4). Structural Equation Modelling (SEM)/path modelling was used in most of the studies (*n* = 15), with 67% of those who used SEM/path analysis reporting the fit of the model. SEM has been found to exhibit superior properties compared to regression analysis in overcoming the limitations of regression by decomposing the sources of correlation among independent variables [21] and make it possible for each variable in a path model to be treated simultaneously as both a predictor and as an outcome.

**Table 2:** Application of Wilson and Cleary model

				Characteristics of Study						
Author Year Country	Population	Design	Latent factors/measure	Sample size	Age Mean (SD)	% of Female	Aim of study	Analytical Tool	Results/Findings	Percentage of variance explained by model
Ade-Oshifogun 2012 USA	Obesity/Chronic Pulmonary Disease (COPD)	Cross sectional	BP: BMI, FEV1, DLCO, Percent trunk fat (DEXA) SS: Dyspnoea (CRQ), fatigue (CRQ), sleep apnoea (ESS) FS: 6-min walk distance (6MWD) GHP: Functional Performance Inventory (FPI)	76	69.7 (10.3)	35.5%	To test a theoretically and empirically supported model of the relationship among clinical variables, symptoms, function status and health status of elderly people with COPD	Path analysis	● Function status, symptoms and biological variable DLCO have direct causal effect on health status ● DLCO ad dyspnoea predict functioning ● The effect of clinical variables on health status is mediated by symptoms ● Symptoms, function status and clinical variable indirectly influence health status	● Model explained 29% of the variance ● Clinical variables explain 29.6% of symptoms ● Clinical variables explained 50.5% of function status
Arnold 2005 Netherlands	1. Chronic Obstructive Pulmonary Disease (COPD) 2. Chronic Heart Failure (CHF)	Cross sectional	BP: COPD: FEV_1_ VHF: LVEF SS: Dyspnoea measured by a questionnaire FS: Physical Functioning subscale of SF-36 GHP: General health subscale of SF-36 HRQL: Perceived health competence scale	COPD:95 CHF 90	65 (9.3) 59 (10)	35.8% 24.4%	To investigate relationship between objective and subjective health in patients with COPD and CHF	Structural equation model (SEM)	● Biological/physiological variables in both diseases are not significantly related to symptoms but predict physical functioning for COPD (β = 0.20) and CHF (β = 0.17) ● Symptoms predict physical functioning in COPD (β = 0.63) and in CHF (β = 0.67). ● Physical functioning associate with general health perceptions in COPD (β = 0.39) and CHF 9 β = 0.32) ● Symptoms directly associate with general health perceptions only in COPD ● In COPD, symptoms, physical functioning explain general health perception ● Only physical functioning explains general health perceptions in CHF	● Global HRQL explained by symptoms and general health perceptions in both diseases.
Baker 2007 UK	Xerostomia	Longitudinal	BP: Salivary flow Clinical signs SS: Xerostomia Inventory (XI) FS: (OHIP-14) GHP: Global oral health rating (GOH) HRQL: (HADS)	85	59.8 (11.5)	76.5%	To systematically test Wilson and Cleary conceptual model of the direct and mediated pathways between clinical and non-clinical variables in relation to the oral health-related quality of life (OHRQoL) of patients with xerostomia.	Structural Equation Modelling (SEM)	● More severe clinical signs were associated with worse patient-reported symptoms ● More symptoms predicted a greater impact on everyday oral functioning ● Worse functioning predicted lower global oral health perceptions ● Both biological indicators and functioning predicted subjective well-being	● Function accounted for 96.9% of total effects ● 88.2% of total effect on functioning was mediated by symptoms status ● Symptoms 9% ● Functioning 22% ● GOH 24% ● Well-being 21%
Brunault 2014 France	Obesity	Cohort	BP: BMI Type of Surgery SS: BDI Bulimic Investigatory Test, Edinburg (BITE) FS: Quality of Life, Obesity and Dietetics (QOLOD) -Physical QoL -Psychological QoL -Social QoL -Sexual QoL -Comfort with food	126	40.2 (10)	79.4%	To put the Wilson Cleary model to test by determining the predictors of postoperative change in each QoL dimension 12 months after bariatric surgery	Linear mixed model	● Improvement in Psychosocial QoL was associated with lower preoperative depression severity, lower preoperative binge eating severity and higher weight loss ● Improvement in Sexual QoL was associated with lower preoperative depression severity, lower preoperative binge eating severity and younger age ● Improved comfort with food was associated with lower preoperative binge eating severity	● ?
Carlson 2014 USA	Heart Failure	Cross-sectional	BP: Number of chronic illness Comorbidity burden (CCI)as in index of severity of illness Diagnosis of diabetes Diagnosis of chronic atrial fibrillation SS: Depression measure with PHQ-9 Physical symptoms measured with KCCQ FS: Physical and social functioning measured with KCCQ GHP: First item in the SF-36(v2)	265	62	35.8%	To determine the key predictors of overall perceived health (OPH)	Hierarchical multiple regression	● Age, gender and race/ethnicity were predictors of OPH ● Perceived sufficiency of income, social functioning, comorbid burden, symptom stability, black compared to white race were independent predictors of OPH ● Physical and social functioning mediated the effect of SOB and fatigue on OPH as well as the effect of symptom on OPH	● 39.2%
Cosby 2000 USA	HIV/AIDS		BP: CD4 counts SS: Health distress, mental health, energy/fatigue and pain of Health Status Questionnaire (HSQ), SSC-HIV FS: Physical, role, social and cognitive functioning of HSQ GHP: QAM, General health perception of HSQ HRQL: Overall quality of life of HSQ	146			To determine the relationships among haematological complications associated with AIDS, characteristics of the individual and the five dimensions of Wilson and Cleary model	Logistic regression	● All five dimensions of Wilson and Cleary model significantly predicted anaemia.	
Eilayyan 2015 Canada	Asthma	Longitudinal	SS: Physical symptoms (MAQLQ-symptoms) Emotional symptoms (MAQLQ-emotion) Self-efficacy (KASE-AQ) FS: Physical function (MAQLQ-activity)	299	62.1 (14.4)	69%	To identify direct and indirect predictors of perceived asthma control among primary care population.	Path model	● Symptom was affected by self-efficacy ● Emotional status was affected by symptom and self-efficacy ● Physical activity was affected through symptom, emotional status and self-efficacy ● Perceived asthma control at baseline was affected by asthma symptom, physical activity, self-efficacy and smoking ● Perceived asthma control at follow-up was predicted by asthma symptom, physical activity, self-efficacy and baseline perceived asthma control. ● Perceived asthma control was indirectly predicted by emotion status through self-efficacy and physical activity	
Halvorsrud 2010 Norway	Chronic Disease	Cross- sectional	SS: Geriatric Depression Score (GDS-15) FS: SF-12 subscale of physical function GHP: Health satisfaction: global item measure from WHOQoL-Bref HRQL: WHOQoL-Old	89	78.6	73%	To explore the predictors of QOL among community-dwelling older adults receiving community health care	Path analysis: Structural equation Modelling (SEM)	● Environment has direct effects on QOL and indirect effects on QOL with depressive symptoms and health satisfaction (GHP) as mediators ● Depressive symptoms had an indirect, negative effects on QOL with physical functions and general health perceptions as mediators ● Health satisfaction was a mediator between physical function and QOL	● The predictor variables accounted for 37% of the variance in depressive symptoms, 29% in physical function, 44% in general health perceptions and 66% of the variance in QOL (the overall model)
Heo 2005 USA	Heart failure	Baseline data	BP: Patient interview Medical records, CCI SS: Patients perception of Presence and severity of dyspnoea and fatigue measured by Dyspnoea-Fatigue Index Questionnaire FS: NYHA GHP: SF-36 HRQL: MLHFQ	293	73 (11)	53%	To determine the bivariate relationships between HRQL and other variables proposed by Wilson and Cleary To determine best multivariate model based on these variables To test specific components of the Wilson and Cleary model of HRQL	Multiple regression	● Health perception, symptom status and age predict HRQL ● Health perception mediates the effect of symptoms on HRQL ● Functional status does not mediate the effect of symptom status on health perception	● Final model explains 29% of the variance
Hofer 2005 Austria	Coronary Artery Disease (CAD)	Longitudinal	BP: Severity of CAD (no of diseased vessel No. of risk factors SS: Canadian Cardiovascular Society classification of angina pectoris FS: SF-36 physical function score GHP: SF-36 general health score HRQL: Scores on the three scales (physical, social and emotional) of MacNew Heart Disease Quality of Life Questionnaire	432	61.8 (10.2)	24.1%	To apply Wilson and Cleary model a priori to patients with CAD in a prospective longitudinal design and to find out whether it is applicable to CAD patients and is stable over time.	Structural Equation Modelling (SEM)	● Physical functioning, anxiety symptoms have effect on overall HRQL ● Anxiety predicts poorer HRQL ● Depression affects physical functioning and general health perception. ● The higher the level of anxiety, the more severe the symptoms reported	● Final model explains 49% at baseline, 62% one month after and 66% 3 months after intervention of the variance of overall HRQL
Kanters 2012 Netherlands	Pompe disease	Cross-sectional	BP: Enzyme activity (fibroblasts) Skeletal muscle strength assessed by MRC, respiratory function assessed by FVC SS: shortness of breath, Fatigue assessed by Fatigue Severity Scale (FSS) FS: Rotterdam Handicap Scale (RHS) GHP: EQ-5D Visual Analogue Scale (EQ-5D-VAS) HRQL: MCS and PCS of SF-36 Utility derived from EQ-5D	103	49.3	50.6%	To develop a conceptual model for Pompe disease in adults and statistically test it in untreated patients	Random effects linear regression	● MRC and FSS were negatively associated with disease duration ● FVC was affected by female gender ● RHS was affected by FSS, MRC, FVC and Age ● EQ-5D Vas was associated with RHS and disease duration ● MCS was associated with EQ-5D VAS ● PCS was associated with EQ-5D VAS ● Utility was associated with EQ-5D Vas	
Krethong 2008 Thailand	Heart Failure	Cross- sectional	BP: Medical records-LVEF SS: Cardiac Symptoms Survey (CSS) FS: NYHA functional classification GHP: 100 mm horizontal visual analogue scale HRQL: MLFHQ	422	58.47	Ns	To develop and test a hypothesized causa model of HRQL in Thai heart-failure patients	Structural equation modelling (SEM)	● Biological/physiological affected functional status (β = −0.34, *p* < 0.05). ● Symptom affected functional status (β = 0.45, *p* < 0.05); GHP (β = −0.27, *p* < 0.05) and HRQL (β = −0.48, *p* < 0.05) ● Functional status had impact on GHP (β = −0.28, p < 0.05); HRQL (β = −0.25, p < 0.05) ● Social support had impact on symptom (β = −0.25, *p* < 0.05); GHP (β = 0.19, *p* < 0.05) and HRQL (β = −0.17, p < 0.05) ● The effect of biological/physiological on symptom was not significant.	Model explained 58% of the variance in overall HRQL
Mathisen 2007 Norway	Heart Surgery	Longitudinal	GHP: General Health subscale of SF-36 HRQL: Global Quality of Life (gQoL) Norwegian version of the Quality of Life Survey (QoLS-N)	108	64.2	19%	To investigate the existence of a reciprocal relationship between patients’ assessment of quality of life and their appraisal of health.	Structural equation modelling (SEM)	● Baseline overall QoL has a cross lagged effect on three months assessment of general health ● The path from general health at six months to QoL at 12 months was significant ● The simultaneous effects model demonstrated a bidirectional causal paths at each point observed after baseline	
Mayo 2015 Canada	Stroke	Cross-sectional	BP: Side of lesion Stroke severity measured with CNS, CCI SS: SIS Pain: SF-36 (body pain) Vitality: SF-36 (vitality) Emotional well-being: SF-36 (mental health) FS: *Physical Functioning*: SF-36 (PF) SIS (mobility) Health Utility Inventory(HUI): HUI (ambulation) HUI (dexterity) *Social Functioning:* SF-36 (SF) SIS 8b *Role:* Worst of SF-36 RE & RP Cognitive: Mini mental State Education (MMSE) GHP: EQ-5D VAS SF-36 (General health)	678	67.3 (14.8)	45%	To empirically test a biopsychosocial conceptual model of HRQL for people recovering from stroke	Structural equation modelling (SEM)	● Less comorbidity, less pain, better memory and more vitality associated with better health perception.	
Nokes 2011 USA	HIV/AIDS	Cross sectional	SS: Centre for Epidemiological Depression Scaled (CES-D) Revised SSC-HIV Body Change Distress Scale HRQL: HAT-QOL	1217	41.7 (9.1)	31.5%	To determine if there were age-related differences in symptoms status and HRQL for HIV-positive persons aged 50 years and older compared with younger (aged 49 years and younger).	Stepwise regression	● Age was a predictor for sexual function and provider trust ● Less depressive symptoms and less body change distress were related to increase in sexual functioning	
Phaladze 2005 Sub-Saharan Africa	HIV/AIDS	Cross sectional	BP: Has been given AIDS diagnosis Has Comorbidities SS: Revised SSC-HIV FS: Overall functioning GHP: Health worries HRQL: HAT-QOL.	743	34.1 (9.6)	61.2%	To increase understanding of the meaning of quality of life for people living with HIV/AIDS in four countries in Sub-Saharan Africa: Botswana, Lesotho, South Africa and Swaziland.	Hierarchical multiple regression	● Daily functioning predicts overall HRQL ● Higher level of education associates with lower HRQL ● Higher symptom intensity associates with lower HRQL ● A close correlation between symptom intensity and functional status	● Overall model explains 53.2% of the variance
Portillo 2005 USA	HIV/AIDS	Cross sectional	BP: Has been given AIDS diagnosis Has Comorbidities SS: Revised SSC-HIV FS: Overall functioning GHP: Health worries (HAT-QOL)	920	41 (8.7)	32.6%	To test the Wilson and Cleary model in a sample of ethnic minority persons living with HIV/AIDS	Hierarchical regression	Association between physiologic factors, symptoms, functioning, general health perception and life satisfaction	● Overall model explains 22.9%
Saengsiri 2014 Thailand	Coronary Artery Disease (CAD)		BP: LVEF Rose Questionnaire for angina Rose Dyspnea Scale (RDS) SS: Centre for Epidemiologic Studies Depression Scale (CES-D) Cardiac Self Efficacy Scale (C-SES) FS: Functional Performance Inventory Short-Form (FPI-SF) SF-36 Vitality subscale HRQL: Quality of Life Index-Cardiac Version	303	61.2 (10.9)	26.4%	To explain relationship between cardiac self-efficacy, social support, biological and physiological (LVEF) symptoms of angina, dyspnoea, depression, vital exhaustion, functional performance and quality of life in post-PCI CAD patients	Pearson Correlation Path analysis	● Social support (β = 0.31), depression(β = 0.24), vital exhaustion (β = 0.23) and cardiac self-efficacy(β = 0.21) had the most powerful direct effect on quality of life of post-PCI CAD patients ● Self-efficacy had indirect effect on quality of life (β = 0.21, *p* < 0.001)	
Santos 2015 Brazil	Oral health	Cross sectional	BP: Edentulism (dentate = 0, edentulous = 1) assessed by clinical examination SS: Assessed using the question, “are you satisfied with the appearance of your prostheses?” FS: Assessed with the question, “have you decreased or changed the type of food because of problems with your teeth or dental prostheses?” GHP: Assessed using the question, “compared with others your age, how would you rate the health of your mouth overall?” HRQL: OHIP-14	578	68 (6.3)	67.3%	To test the Wilson and Cleary model of the direct and mediated pathways between clinical and non-clinical variables in relation to oral health-related quality of life	Structural Equation Modelling (SEM)	● Dissatisfaction with symptom status are associated with worse functional status ● Worse functioning predicts poor oral health perception ● Poor oral health perception associates with higher worse oral health quality of life ● Final model shows negative significant direct effect between biological variable and symptom status ● Age, gender and geographical location have direct paths to biological variable (edentulism) ● Age and gender directly impact oral health-related quality of life	● The comparative fit index is 0.98 indicating adequate fit.
Schulz 2012 Netherlands	Kidney Transplant	Cross-sectional	BP: Number of active comorbidities reported by patients FS: European Quality of Life −5 dimension (EQ-5D) GHP: EQ-5D Visual Analogue Scale (EQ-5D-VAS) HRQL: General Health Questionnaire (GHQ-12)	609	53.7 (12.3)	43.9%	To identify pathways through which objective health affects psychological distress and to clarify how personal characteristics are shaped by objective health and determine psychological distress	Structural equation modelling (SEM)	● Impact of objective health and functional status on psychological distress was fully mediated by subjective health and personal characteristics ● Influence of objective health was mediated by successively by functional status and personal characteristics; successively by functional status and subjective health; exclusively by personal characteristics and; exclusively by subjective health	The model explained 32% of variance of psychological distress
Shiu 2014 Hong Kong	Diabetes	Cross sectional	BP: Time since diagnosis Age of onset and type of diabetes HbA1c level, blood pressure and lipid profile SS: Self-reported comorbidity characteristics and presence of comorbidity and no of comorbidities FS: Physical functioning subscale of SF-36 Older American Resources and Services Multidimensional Functional Assessment Questionnaire GHP: SF-36: general health Self-developed ratings 6 HRQL: subscales of the SF-36: role-physical, role-emotional, mental health, social functioning, bodily pain and vitality	452	71.8 (7.3)	59.1%	To apply the Wilson and Cleary model of HRQL to understand the relationship among clinical and psychological outcomes in community-dwelling older Hong Kong Chinese people with diabetes.	Structural Equation Modelling (SEM)	● Four determinants: general health perception, psychological distress, adequacy of income and social support have direct effect on HRQL ● Three determinants: symptom status, physical functional status and psychological status have indirect effects on HRQL through general health perception ● Four determinants: symptom status, age, gender and physical activity have indirect effect on HRQL through physical function status	● The model explains between 64% and 72% of variance
Sousa 1999 USA	HIV/ AIDS	Cross- sectional	BP: APACHE III SS: HIV-problem checklist FS: HIV Quality Audit marker (QAM) GHP: MOS-30 (single item for GHP) HRQL: MOS-30 (single item for overall quality of life	142	38 (8.7)	20%		Multiple regression	● Symptoms correlated negatively with GHP (*r* = −0.48) and overall HRQL (*r* = −0.37). Functional status positively associated with GHP (*r* = 0.22) and overall HRQL (*r* = 0.29) Biological/physiological variables do not have significant associations either directly or indirectly on any of the variables.	●
Sousa 2006 USA	HIV/ AIDS	Cross- sectional	BP: CD4 Count SS: SSC-HIV FS: The Health Assessment Questionnaire-Disability Index (HAQ-DI) GHP: 100 mm visual analogue scale Ordinal scale HRQL: Derived from general health status scales	917	30.4 (8.13)	43%	To estimate the primary pathways of the Wilson and Cleary HRQL conceptual model using structural equation modelling (SEM)	Structural equation modelling (SEM)	● A significant relationship between status and functional health (*r* = 0.56) ● There is significant relationship between symptoms status and general health perceptions (*r* = −0.33) and functional health and general health perceptions (*r* = −0.42) ● There is significant relationship between symptoms status and overall quality of life (*r* = −0.20) and between GHP and overall quality of life (*r* = 0.26) CD4 count had a negative relationship with symptom status (*r* = − 0.20, *p* < 0.05)	● Symptoms explain 49% of functional health ● Both symptoms status and functional heath accounted for 62.5% of the variance of general health. ● Both symptoms status and general heath perceptions accounted for 38,2% of the variance in overall quality of life.
Ulvik 2008 Norway	Coronary Artery Disease (CAD)	Cross- sectional	BP: Myocardial disease LVEF SS: Angina (AFS, CCS) Dyspnoea (NYHA) Anxiety (HADS) Depression (HADS) FS: Physical function Social function GHP: General health (SF-36) HRQL: Overall QoL: measured with a single question	753	61.7 (10.2)	26%	To analyse relationship between disease severity and both mental and physical dimensions of HRQL.	Linear and ordinal logistic regression	● Biological variables associate with symptoms ● Depression associates positively with LVEF ● Symptoms affect physical function ● Social function is low in patients with more symptoms of anxiety. ● General health is negatively related to anxiety and depression but positively related to physical and social functions ● Better overall QOL is associated with less symptoms and depression but related negatively to social function	● The model explains 43% of the variance of overall quality of life.
Wettergren 2004 Sweden	Hodgkin’s Lymphoma	Cross sectional	BP: Disease stage (I-IV) Treatment modality (irradiation, chemotherapy or combined modality treatment Time since diagnosis SS: (SEQoL-DW) HADS FS: Measured as part of general health perceptions GHP: PCS of Short Form 12 (SF-12), MCS of SF-12 HRQL: QoL index of (SEQoL-DW)	121	45 (median)	45%	To evaluate HRQL in long-term survivors of |Hodgkin’s lymphoma (HL) and to identify determinants of HRQL using Wilson and Cleary’s conceptual model with the potential goal of improving care and rehabilitation.	Partial Correlations	● Disease stage correlated with Disease index (SEQoL-DW) ● Lower SOC was related to a worse HRQL ● Poorer physical health was associated with worse overall quality of life.	
Wyrwich 2011 USA	General Anxiety Disorder (GAD)	Longitudinal	BP: CGI-S SS: HAM-A FS: PSQI GHP: Q-LES-Q(SF) (items 1–14) HRQL: Q-LES-Q(SF)) (Item 16)	1692	40.3 (11.8)	65.1%	To test the application of the Wilson-Cleary model to patient population with generalised anxiety disorder (GAD) using longitudinal clinical trial data.	Path Model	● CGI-S had a strong relationship with HAM-A ● HAM-A at week 8 had strong path (β = 0.5) to PSQI and moderate effect (β = −0.40) on Q-LES-Q(SF) ● Q-LES-Q(SF) had a strong relationship with overall quality of life (β = 0.66)	● Model explained 56% at baseline and 69% at week 8

*DLCO* Carbon Monoxide Diffusing Capacity, *FEV1* Forced Ejection Volume, *FVC* Forced Vital Capacity, *PSQI* Pittsburgh Sleep Quality Index, *LVEF* Left Ventricular Ejection Fraction, *QAM* Quality Audit Marker, *CCI* Charlson Comorbidity Index, *OHIP-14* Oral Health Impact Profile, *KCCQ* Kansas City Cardiomyopathy Questionnaire, *MCS* Mental Component Summary, *BDI* Beck Depression Index, *PHQ-9* Patient Health Questionnaire, *HAM-A* Hamilton Rating Scale for Anxiety, *MRC* Medical Research Council, *CNS* Canadian Neurological Scale, *SIS* Stroke Impact Scale HAT-QOL, *HADS* Hospital Anxiety and Depression Scale, *BMI* Body Mass Index, *PCS* Physical Component Summary, HSQ: Health Status Questionnaire, *CRQ* Chronic Respiratory Disease Questionnaire, *MLFHQ* Minnesota Living with Heart Failure Questionnaire, *NYHA* New York Heart Association, *SEQoL-DW* Schedule for the Evaluation of the Individual Quality of Life Direct Weighting, *CGI-S* Clinical Global Impression-Severity of Illness, *Q-LES-Q(SF)* Quality of Life, Enjoyment and Satisfaction Questionnaire-Short Form, *HIV/AIDS* Targets Quality of Life, *SSC-HIV*-Signs and Symptoms Checklist for Persons with HIV/Disease, *WHOQOL* World Health Organisation Quality of Life

### Research question 1: Does empirical evidence show the causal relationship of the dominant concepts as proposed in Wilson and Cleary’s model?

#### Adjacent linkages and mediators

**Fig. 2:**
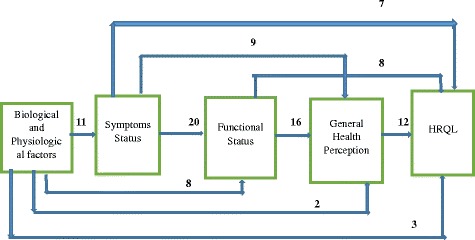
Adjacent and non-adjacent linkages of concepts

Wilson and Cleary [10] hypothesised that there existed direct causal links between biological and physiological factors, symptoms, functional status, general health perceptions and HRQL. Symptoms mediate between physiological factors and functional status, while functional status mediates between symptoms and general health perceptions, and general health perceptions mediates between functional status and overall HRQL. Eleven studies supported the direct causal link proposition between biological and physiological factors and symptoms (Fig. 2). Markers of biological and physiological variables were found to associate with worse symptoms in the patients with HIV/AIDs, xerostomia, coronary artery disease, Hodgkins lymphoma and generalised anxiety disorder [17, 37, 38, 40, 46]. The next level of the model associates symptoms with functioning and mediates between functioning and biological/physiological variables. This has been established in 20 studies (e.g., [16, 22, 29]). More symptoms predicted a greater impact on everyday functioning, with symptoms status explaining 49% of functional health in HIV/AIDs patients [37]. Functional status was found to have direct links to general health perception and mediated between general health perception and symptoms in 16 studies. Worse functioning indicated low perceived health. For example, worse functioning was associated with lower global oral health perception in Hodgkin’s lymphoma [46]. More symptoms and less functional health may lead to a perceived decrease in perceived general health. The hypothesised effect of general health perception on overall HRQL was established in 12 studies (Fig. 2).

### Research question 2: Does the Wilson and Cleary model strictly follow a linear unidirectional path?

#### Linkages between non-adjacent concepts

We examined the links between non-adjacent variables to establish whether empirical data show that the model allows non-linear, indirect paths.

Biological and physiological variables were directly associated with functional status, general health perception and overall HRQL in nine, two and three studies respectively (Fig. 2). For example, Kanters et al. [25] showed that enzyme activity, a biological marker, was significantly associated with HRQL in adult Pompe disease. Direct links were established between symptom status and, general health perception and HRQL in nine and seven studies respectively. Furthermore, functional status was associated directly with overall HRQL in seven studies. In coronary artery disease, physical functioning showed high positive significant effect on HRQL (β = 0.36) indicating that a reduction in functional health may reduce HRQL [24]. The studies assumed non-reciprocal relationships except Mathisen et al. [27] who attempted to model reciprocal relationship between general health perception and HRQL. This did not take into consideration possible effects between other concepts. Hence, we could not establish the possibilities of bidirectional relationships between the abstract concepts in this study.

### Research question 3: What is the relative effect of each variable?

**Fig. 3:**
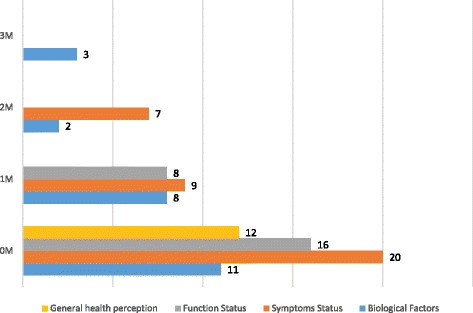
Bar chart of observed magnitudes of effects

The relative effects of the variables were measured in terms of the magnitude of their influence on HRQL (Fig. 3). The causal links were labelled 0 M, 1 M, 2 M and 3 M to signify the number of mediators between constructs that were bypassed. 0 M was a direct link between the concepts with the proposed mediating variable signifying that no mediator was bypassed in the link, 1 M was an indirect link with one mediator bypassed, 2 M with two mediators bypassed and 3 M with three mediators bypassed. 0 M, 1 M and 2 M revealed symptoms status as a consistently important factor that affected HRQL, followed by functional status. In 0 M all four concepts; biological and physiological, symptom status, functioning status and general health perception were compared with respect to the effect of each on the adjacent variable. Clinical variables had the lowest magnitude of effect followed by general health perception, function status and symptoms status in order of increasing magnitude. Clinical factor was however on the same level with functional status when only the immediate mediator was bypassed.

## Discussion

### Brief summary

The findings of this systematic review support the model of HRQL as proposed by Wilson and Cleary [10] and establish the conceptualised relationships and mediation. Of the articles reviewed, 74% found symptom status a significant predictor and critical mediator making it the most important predictor of HRQL by indirect effects through functional status and general health perceptions and by direct effects. More symptoms implied impaired functioning with consequently worse general health perception and lower HRQL. Most of the studies (73%) were of moderate quality; this was because the quality assessment criteria [13] gives low ratings to study designs that are not experimental or longitudinal in nature.

### Comparison with previous studies

Both symptoms and general health perception were found to account for 38.2% of variance in global HRQL [26] and studies also showed that general health perception alone mediated the relationship between symptom status and HRQL [16, 28, 35, 37, 38]. One of the most important symptoms was depression which strongly associated with physical functioning (β = −0.32) and general health perceptions [24]. Two studies found no association between the clinical factors, and any of the health constructs in heart failure and diabetes [23, 35]. This may be due to other profound non-clinical factors that responsible for impaired HRQL in these populations. The non-adjacent links among the health concepts showed that the model was non-linear also the effects of the variable were not fully mediated by their proposed mediators. For example, the direct link between symptom status and overall HRQL indicated that both functional status and general health perception did not fully mediate the effects of symptom burden on HRQL.

### Possible explanations and implication

The findings of Sullivan et al. [14] in coronary artery disease patients supported our findings of a direct link between biological and physiological variables, and functional status. Further studies of a longitudinal nature will be required to establish possible bidirectional relationships among the concepts and whether the factors exert reciprocal influence on each other. For example, while the symptoms of pain may reduce functioning in patients with sickle cell disease, the inability to function as expected may lead to depression which may further limit functioning and lower HRQL. Our study is the first to synthesize results of studies on Wilson and Cleary’s model and to establish the relative importance of the constructs in determining the quality of life of patients in chronic diseases.

## Limitations

We identified some potential limitations to this study. The study focused on several chronic diseases which have different clinical statuses, prognoses and levels of disability, which restricts our ability to generalise based on the lack of homogeneity of symptom status and functional status of the patients. There is also the potential limitation due to publication bias as only published articles were used in this study. In addition, different instruments were used to measure HRQL in the included studies; while some are generic, some are disease-specific [47]. As there is no instrument that is a “gold standard”, researchers often select instruments sensitive to the health state they are investigating ([48] rather than a general measure of HRQL. Moreover, there are also variations in clinically important differences across groups of patients defined by diseases, conditions, severity level, socio-economic status and nationality [49].

## Conclusion

Our findings show that the Wilson and Cleary model demonstrates a good fit and proved useful in identifying relationships among the health constructs, and predictors of HRQL in the studied disease populations. The model explained between 22.9% and 72% of the variance in overall quality of life indicating that, in some cases, the model may require modification to capture factors not specified in the model but that may be important determinants of overall quality of life.

The findings supported the robustness of the Wilson and Cleary model as a conceptual framework to characterise predictors of HRQL in chronic diseases and to aid understanding of the relationship between clinical and psychological outcomes for patients with chronic illness. Our understanding of specific directions of influence will aid healthcare practitioners and researchers to develop appropriate care protocols that will address psychosocial variables alongside clinical factors in chronic disease management. This study has demonstrated that symptoms are a major determinant of HRQL in patients with chronic disease, thus a clinical approach to reduce symptoms may help improve HRQL. Furthermore, in treating patients with chronic diseases, clinicians and healthcare practitioners should be alert for signs of depression because this study has highlighted depression as a major issue in HRQL.

Further work is needed to examine bidirectional relationships. Studies so far have focused on an assumption of no reciprocal relationship but low health perception or low HRQL might also worsen disease conditions and responses to treatment. Further studies on evaluation of the Wilson and Cleary model should be compared to the findings of this study.

## Abbreviations

HRQL: Health-related quality of life.
PRISMA: Preferred Reporting Items for Systematic Reviews and Meta-Analysis
SEM: Structural Equation Modelling
